# A Treatise on the Venereal Disease and Its Varieties

**Published:** 1833-07-01

**Authors:** 


### II. *(bis.)*

A TREATISE ON THE VENEREAL DISEASE AND ITS VARIETIES. By *William Wallace*, M.R.I.A. &c. Surgeon to the Jervis Street Infirmary, Dublin, and to the Infirmary established in that City for the Treatment of Cutaneous Diseases, including Venereal Diseases. Octavo, pp. 382. London, 1833.

THERE can be no question of the want of a good description of the venereal disease, and, unfortunately, there can be as little doubt of the difficulties connected with an attempt to supply that want in a satisfactory manner. The history of the disease is a history of the most contradictory opinions that have ever been entertained, even in medicine. Authority is pitted against authority, and, strange to say, this war of dogmas has not been productive of freedom of thought and of accurate investigation, but has ended in a submission, as slavish as disgraceful, to the despotism of a name. It is time to throw off the yoke of Mr. Hunter, and when we have freed ourselves from the fetters with which his hypothetical and fanciful mind has bound us, we may discover, in rigid observation and induction, an approach to the certainty and truth that have hitherto altogether eluded us.

Those who have had an opportunity of studying extensively, and have employed their opportunities in observing carefully, the venereal disease, must be too well convinced that much general ignorance exists with respect to its nature, and that much mischief is perpetrated in its treatment. We have this day seen a patient, who has been subjected to a course of mercury of five months' duration for a primary sore, with the effect of producing almost total destruction of the soft parts of the nose, and a great amount of injury to the constitution. This most injudicious treatment was not from the hands of a quack, or an otherwise ignorant person, but from one representing very fairly the ordinary attainments of professional men.

A tolerable knowledge of the sores and the forms of eruption that do or do not require or bear mercury, is certainly not to be found in the generality of medical practitioners. They do not appear, and we speak from what we have witnessed, to possess any definite notions respecting either the disease or its treatment—to be aware of the varieties of constitutional and local symptoms, and the local or general states giving rise to or accompanying those varieties—they wield mercury as a madman would a sword, wildly and destructively. This is not idle fanfaronade, but sober and melancholy truth, and the writer who can remedy this deplorable condition of things, who can lead the mass of the profession to more accurate notions of the phenomena of the disease, and a more appropriate and definite application of remedies, will deserve well of mankind.

Our limited space forbids us to indulge in lengthened prefatory observations, and we shall proceed at once to the review of the work before us. We should mention, however, that Mr. Wallace has enjoyed considerable opportunities for investigating the subject, and that he appears to have begun and conducted the investigation in a rational and philosophical manner. Having diligently observed the cases which hospital practice presented to him, he was soon impressed with the fact, that books and nature disagreed. He shall relate in his own words the method he then adopted, as we feel assured that, whether Mr. Wallace has succeeded or not, it is the only mode of arriving at any important and satisfactory conclusions in the study of this malady.

"But I soon found it impossible to reconcile the writings of the best authors on the venereal disease with many of its most common phenomena; and it appeared evident, that if I wished to make any very satisfactory progress in its study, it would be necessary to unshackle my mind from authority, and to take nature only for my guide. In fact, there seemed to be no alternative, but either to investigate the subject *de novo*, or to remain content with a very imperfect knowledge of a disease which daily obtruded itself on my observation, and with which it was therefore of the first practical importance to be intimately acquainted. The former alternative I preferred, and with the object of carrying it into effect, I commenced, so long ago as the year 1819, when an additional field for observation was opened to me by my election as Surgeon to the Jervis Street Infirmary, to note every case of venereal disease which occurred, but with particular accuracy such as presented any interest or peculiarity, and at the same time to collect, with the assistance of qualified artists, accurate delineations, not only of all the varieties of this disease, but of all the phases of each variety.

During the earlier period of this investigation a system of treatment the least likely to interfere with the operations of nature, was as far as possible adopted, with the object of acquiring a knowledge of the natural history of the disease;

—the local applications being in general, lint wet with water, and, when necessary to prevent evaporation, covered with oiled silk, or with a pledget of wax ointment; while all constitutional remedies, except mild laxatives, were avoided, unless when the patient's safety required, from the supervention of alarming symptoms, more active measures; and these were then employed in conformity to the general principles of medicine and surgery, totally abstracting from consideration every idea of the disease possessing specific characters, or requiring a specific course of treatment. After this practice had been pursued for a sufficient time to fulfil the objects in view, various other plans of treatment, suggested by previously acquired knowledge of the advantages and disadvantages of mercury, &c. were tried, and by degrees that recommended in the following pages was adopted, from a full conviction of its being, on the whole, the most secure and satisfactory.

By a steady perseverance for some years in the plan of investigation just mentioned, I accumulated, at great expense, and with much labour, a collection of representations and descriptions of venereal diseases, so numerous and varied, that at last I ceased to meet any form, or even any stage of these affections, in either hospital or private practice, which was not frequently described in my case-book, and often represented among my drawings.

When this period arrived, my next object was to arrange the great mass of materials thus collected, so as to enable me to arrive at such general conclusions as the facts observed would warrant. But, in consequence of the objects being extremely numerous, and apparently most heterogeneous and discordant, their classification was found a much more difficult task than might be supposed. However, after examining and comparing, and arranging in groups over and over again the delineations, which with my notes recalled a vast assemblage of cases almost as forcibly to mind as if they were present—just as the *hortus siccus* of a botanist vividly reminds him of the more attractive objects of his study—a natural classification of venereal diseases at last occurred to me; and, following up my first notions on this important subject, it became evident, that there existed among all these affections the closest analogy; and that, although greatly diversified in their appearance, they are referable to one regular form of disease, and to a limited number of varieties, as fixed in their characters and relations as the varieties of any other disease."—*Preface*, vi.

Mr. Wallace was in London some two years ago, and we then had an opportunity of inspecting the drawings of the various forms of the disease, which he had procured from artists expressly engaged for that purpose. If published on a scale commensurate with their value, they will redound in an equal degree to the credit of the person who could entertain the design, and advantage of the public that should encourage its execution.

The first volume of the work, and this only is at present brought forward, is devoted to the consideration of the primary symptoms of syphilis, and bubo. The succeeding volume, which will be published at an early opportunity, will embrace the secondary symptoms, and the plates, which will be laid before the public in periodical fasciculi, will complete the whole. The first chapter of the present volume is occupied with the discussion of two general questions:—First, Are venereal diseases produced by a specific cause or morbid poison; or do they arise from common causes of irritation? Secondly, Are venereal diseases produced by one poison, or by a plurality of poisons? For reasons which we shall explain hereafter, we shall waive these inquiries for the present, and pass to the second chapter, which treats of the morbid states or actions produced by the direct application of the venereal poison.

Mr. Wallace is inclined to believe that the followers of John Hunter attribute to him opinions too exclusive on the subject of chancre, or the ulcerative form of primary syphilis. At page 215 of his work he remarks: "Venereal ulcers have *commonly* one character;" "a chancre has *commonly* a hardened base." The disciples of Hunter, however, attributing a certain collection of features to a syphilitic sore, and finding the sore so painted and so imagined, uncommon in the present day, conclude that the syphilis, which they assert was imported from America, is now seldom to be met with. Mr. Wallace justly observes, that supposing the case to be as stated, that Mr. Hunter did intend his description of chancre to be that of the true syphilitic sore, we are bound to reject that description if we do not find it borne out by facts; in other words, the contest lying between Mr. Hunter and Nature, we must in preference believe the latter. Of this there can be no doubt, and we trust that the day is past, for the authority of any man to command implicit and blind obedience.

Another fallacy, which in Mr. Wallace's opinion has materially retarded the advance of our knowledge of the venereal, is the old and deeply-rooted prejudice, that mercury is indispensable to the cure of syphilis. Those who entertained that opinion, finding it impossible to resist the facts that have been of late years brought forward, have been forced to fly for refuge to the fable, that the disease which indispensably requires this remedy no longer exists, and that the present fry of venereal complaints are not the genuine syphilis.

The remainder of the chapter is occupied with, *first*, an enumeration of the morbid states or actions produced by the direct application of the venereal poison; and, *secondly*, a classification of those morbid states and actions. We are averse to dealing with general questions at present, and yet we do not see how we can avoid these sections, without impairing the intelligibility of the subsequent portions of the work. We shall be as brief as possible.

### Morbid States and Actions produced by the direct Application of the Venereal Poison.

Mr. Wallace observes, that if the venereal poison or those secretions which contain it are applied to a susceptible surface, there results, sooner or later, an accumulation of blood, and this is quickly followed, preceded, or accompanied by—1. Inflammation, which varies in degree, and may subside, leaving the part in a state of integrity, or lead to other morbid states: 2. A diminution of the extensibility, or perhaps a state of morbid contraction of the diseased tissues, from which phymosis, paraphymosis, &c. may result: 3. An increased and altered secretion into the subjacent parts, producing either the states of œdema or induration: 4. An increased and morbid secretion from the diseased surface, constituting the state commonly called gonorrhœa: 5. Excoriation, or removal of the cuticle, with a consequent morbid discharge from the denuded surface: 6. Ulceration, or the removal of more or less of the parts subjacent to the cuticle, attended, like excoriation, by a morbid discharge from the denuded and diseased surface: 7. Mortification or sloughing.

Our readers will, from this enumeration, perceive the grounds of our dislike to commence with general considerations. These if worth any thing, should be the expression of the detailed facts, or the sum of the special reasonings founded on facts advanced. But the reasonings and the facts are not yet before our readers, and, if we dissent from the author, we judge him before his case is fully stated—if we give his sentiments unchallenged, we seem to yield implicit assent to them. In the present instance Mr. Wallace makes the "venereal poison, or those secretions which contain it," a sentence, by the way, that seems tautological, to produce indifferently inflammation which ends in resolution, phymosis, excoriation, sore, or sloughing. We confess that we feel startled at this commencement, and we find it difficult to yield our assent to a doctrine that makes affections of so very different a character equally dependent on the venereal poison. The reasoning appended to the foregoing enumeration of the effects of the venereal poison is as bold as the enumeration itself.

"When the venereal poison is applied to a mucous surface, its local effects are generally limited to the morbid states, 1, 2, 3, and 4. Whereas, from its direct action on a cutaneous surface, one or more of the states 5, 6, and 7 result, in addition to the states 1, 2, and 3.

Now on reflection it would appear, that all the various effects produced by the action of the venereal poison are but links of the same chain; for the states 1, 2, and 3 are common to the states 4, 5, 6, and 7; while we find that the state 4 is a transition to 5, 5 to 6, and 6 to 7.

Although the phenomena exhibited by the states 5, 6, and 7, or excoriation, ulceration, and mortification, are in appearance very different, a little reflection will convince us, that in their intimate nature they are either the same or quite analogous. In fact, these states are all preceded by a morbid action, somewhat resembling inflammation, followed by an alteration of structure of the part, preparatory to its removal which sooner or later inevitably occurs, as the alteration it has undergone renders it unfit to remain longer in union with the body." 47.

This appears to us to be extraordinary reasoning. Because inflammation and its consequences, contraction and serous or solid effusion, are "common to" gonorrhœa, excoriation, ulceration, and mortification, therefore all are but links of the same chain, and all the result of a common poison! Mr. Wallace will pardon us when we observe that the argument appears to us to be obviously illogical and unsound. All the states which he has mentioned, excepting for convenience-sake gonorrhœa, are or may be the result of common inflammation, and do not per se imply the operation of any poison at all.

It is not the mere fact of there being an ulcer that proves the previous agency of a poison, but other phenomena connected with that ulcer, and succeeding it. If these phenomena are observed, they constitute the evidence of the application of poison, if not, the ulceration itself offers none. To prove the origin of phymosis from poison, we require precisely the same sort of evidence, that of attendant and consequent phenomena, which demonstrates the origin of an ulcer from poison. If that evidence be wanting, the conclusion is not warranted. But, says Mr. Wallace, inflammation attends ulceration, or phymosis attends sore, therefore, inflammation has the same cause as sore. This is obviously reasoning in a circle, and a very small circle too, and an analogical case will shew its fallacy. Inflammation of the Schneiderian membrane attends measles—the latter always arises from a specific contagion, and, if Mr. Wallace's argument be worth anything, inflammation of the Schneiderian membrane must always arise from a specific contagion. We are not aware of having in the least misrepresented Mr. Wallace's argument, nor do we think we have pushed our reduction of it too far.

Again, Mr. Wallace remarks, that the state of gonorrhœa is a transition to excoriation—excoriation a state of transition to ulceration—and ulceration a state of transition to mortification. Thus mortification is an extreme degree of gonorrhœa, for if that be the first of a series of transitions, of course the last in the series is but the Ultima Thule of the first. But even if this were true, and it is palpably untenable, the argument would still be a logical fallacy. It is not, we repeat, the local action that, in the case of syphilis, constitutes the evidence of the application of a special poison—it is the tendency to constitutional contamination, and the consequent development of ulterior symptoms. If this be observed after gonorrhœa, as after an ulcerative or a sloughing sore, the singularity or identity of the producing agency is proved; if it be not observed, the circumstance of the local phenomena displaying transition states, even if it were true, proves nothing, save that the transition was, somewhere, so complete as to constitute an utter difference.

It may be said that by employing the term "transition" states, Mr. Wallace does not intend to affirm, that they differ merely in degree. But he has observed that the three first conditions are "common to" the four last, and that implies that they do not totally or greatly differ. The idea of transition states is after all a very strange and a very indefinite one. What, for instance, can be learnt or understood from the assertion that gonorrhœa is a transition state between phymosis and chancre, or between induration and mortification? We may be deficient in the faculty of apprehension, but we confess ourselves unable to see the clearness of Mr. Wallace's reasoning, or the grounds of his arrangement.

Mr. Wallace enters into an elaborate argument to shew that the loss of substance which attends ulceration is not attributable to the action of the absorbents, but that the process is more akin to that of sloughing. Mr. W. affirms that a change takes place in the part about to be removed, whether by ulceration or by sloughing, the only difference being, that in the former the change is less perceptible and the rejected portion liquified, whereas in the latter the separation occurs in thick and visible portions more or less solid. As this is in some measure an abstract question, we shall waive its consideration, but we would venture to remark that, although the onus probandi is on Mr. Wallace, we do not think his arguments conclusive. This however, is mere opinion, and as our readers are not presented with Mr. Wallace's facts and reasoning's, we can claim no right to pronounce upon them.

It is fortunate for the elucidation of truth, that those who entertain peculiar opinions on subjects difficult of proof, have an opportunity of strengthening them, if correct, or finding the difficulty of supporting them if incorrect, in their exposition and application. Take, for instance, the following passage.

"Were Mr. Hunter's opinions of the process of ulceration correct, as in his judgment ulceration implies absorption, contamination of the system should always follow ulceration; but, as we know that this is not the case, we might almost infer, from this circumstance alone, that ulceration is not caused by absorption. Mr. Hunter was himself well acquainted with the fact, that contamination of the system did not necessarily succeed a chancre, and therefore he admitted that a chancre was a local sore; but, in making this admission, he evidently contradicted his own hypothesis respecting ulceration; for if this process be an act of the absorbents, it is clear, as has been just said, that from the moment at which ulceration first commences, absorption, and consequently contamination of the body, also commences; unless we admit that the venereal poison may be received into the mass of circulating fluids, without necessarily causing constitutional disease,—an admission however which we are not warranted in making.

On the other hand, according to the opinion here entertained of the process of ulceration, it is evident that a chancre may exist without absorption, and therefore without contamination of the system. I would even go farther, and affirm, that it is extremely probable, that so long as the process of ulceration continues, no absorption can take place from the ulcerating surface; and, consequently, that contamination will not occur, until after this process has terminated on either a portion or the entire of the diseased surface. We know that the state of inflammation precedes and accompanies ulceration, and that the action of the absorbents of inflamed tissues is either suspended altogether, or very greatly diminished. To this we may add, that it is extremely improbable that the absorbents of a tissue, either in the state or passing into the state of ulceration or sloughing, are in a fit condition for performing the functions of absorption. It must at least be admitted to be very unlikely that any substance applied to the surface of a sore can be absorbed during ulceration, if we reflect that all ulcerating surfaces are formed of a tissue which is *in transitu* from the state of a living texture to that of an inorganic fluid; and it is quite possible, that although this substance may form a stratum almost inconceivably thin, it may still be sufficient to obstruct absorption.

It will no doubt be affirmed, in opposition to this opinion, that absorption from the surface of ulcers, and consequent impregnation of the system, is notorious. Thus, salivation from mercurial dressings to sores, very frequently occurs. But will it not be found that in all such cases the process of ulceration has actually ceased, on the whole or on part of the surface of the ulcer; and that the surface from which the absorption has taken place is a granulating and not an ulcerating surface?" 53.

In the passage we have extracted there is more than one fallacy, and there are many difficulties. And first for an example of a fallacy. It is not correct to state that if ulceration implies absorption, contamination of the system should always follow ulceration. This presupposes two things, neither of which is proved; first, that the ulceration is always the result of a specific poison; and, secondly, that if the poison be absorbed the constitution must necessarily display evidence of contamination. With reference to the first point it is certain that the slightest excoriation may, if injudiciously irritated, become a troublesome ulcer; and with respect to the second, we may observe, that as all are not susceptible of small-pox, even when the poison is directly applied, so there is no inconsistency in supposing that all are not susceptible of syphilis, and the contrary has not been proved.

The difficulties attending Mr. Wallace's theory of the absorption of the syphilitic poison appear to us to be more formidable than those of the notion he has rejected. If ulceration be a process similar to sloughing, it is difficult, nay impossible, to conceive how the poison can linger in the sore, and only be absorbed after the ulcerative process has ceased. The venereal poison is applied to the prepuce, a sore ensues, that sore is dressed and washed frequently, its surface is thrown off a greater or less number of times, or to a greater or less depth, and yet after all this is over the poison is for the first time carried into the system. Where the poison lurked, and how and why it should operate now, are circumstances which are incomprehensible to us. Setting aside this important consideration, we are presented with no proof that the poison only acts on the constitution after the cessation of the ulcerative action. To say that it is unlikely that absorption can take place during ulceration, because the ulcerating surface is formed of a tissue which is in transitu from the living to the inorganic state, is clearly a petitio principii. Those who are not inclined to grant an unproved premiss, will probably not be disposed to grant a conclusion drawn from it. All questions of this kind must be decided by facts, not by an ingenious accumulation of suppositions, some more some less susceptible of proof. Mr. Wallace argues that it is extremely improbable that the absorbents of a tissue either in the state, or passing into the state of ulceration or sloughing, are in a fit condition for performing the functions of absorption. We have no hesitation whatever in replying, that however improbable this may seem, it is undoubtedly the fact. If. arsenic be applied to a sore, the constitution will be specifically affected, at the very time that the arsenic is acting as a local escharotic, and occasioning sloughing. We have many times seen the oxymuriate, applied in the form of ointment, produce a high degree of local inflammation, ulceration, and even sloughing, whilst the constitution was becoming mercurialized.

Mr. Wallace draws from the views to which we have been adverting, the following practical conclusions :—

"1. If by any means the poisonous quality of an ulcer, produced by the direct application of the venereal virus, can be destroyed, before the process of ulceration has ceased in any point of the ulcer, the contamination of the system will be prevented.

2. If from the violence of inflammation a process of sloughing commences in a chancre before the action of ulceration has ceased upon any portion of its surface, and if this process involves the structure of the part beyond the point of contamination, it may form not only a natural cure of the local disease, but may also prevent contamination of the system. Gangrenous or sloughing chancres afford common examples of the truth of this assertion ; and it should be observed in support of this opinion, that it is admitted, that when the gangrenous or sloughing process attacks venereal sores in their earlier stages, it much more frequently prevents contamination of the system, than when this process does not commence until after the chancre has been for some time in existence.

3. Should a bubo occur, in consequence of a chancre, before the ulcerating process of that chancre has ceased, it is more likely that such a bubo has been produced by irritation, than by absorption of the virus. Indeed, this fact is tacitly admitted by those who have most experience in venereal complaints ; for it is allowed, that buboes are most apt to occur after a lapse of some time from the formation of a chancre ; and it is also admitted, that the longer a chancre has continued before a bubo has been produced, the more likely is such a bubo to be a forerunner of constitutional symptoms. In fact, it is more than probable, that a bubo cannot occur from absorption, until the process of ulceration has ceased in a greater or lesser portion of a chancre ; and if any tumefaction of the neighbouring glands should be observed prior to this period, we may perhaps fairly conclude, that it has been produced by sympathetic irritation. At the same time, the great difficulty which will be always found in distinguishing between a surface, upon which the process of ulceration has not ceased on any possible point, and that upon which this process may have partially ceased, will always prevent a prudent practitioner from acting upon such views with too much security." 54.

To us some of these practical conclusions appear to be rather in the teeth of his doctrines, than fairly deduced from them. But we must pass on. We are next presented with a—

*Classification of the Morbid States and Actions produced by the direct Application of the Venereal Poison.*

Mr. Wallace observes that the several morbid states or actions enumerated already are generally collected into certain groups of symptoms, which have been referred to one of two classes of disease—gonorrhœa or chancre.

" However distinct from each other the chancrous and gonorrhœal forms of venereal disease may appear to be, it is always to be remembered, that they are only modifications of the same specific morbid action, arising from the same exciting cause, and not different species of disease,—consequently, we shall not be surprised to observe, that they are sometimes combined, or that they reciprocally produce each other, or that their influence on the constitution may be similar. And while we admit that this aptitude to combination, reciprocal causation, and similarity of consequences, are all powerful arguments in support of the doctrine of identity of original cause, or of the unity of the venereal poison, we must also admit them as a proof, that a classification of venereal diseases, under the heads of gonorrhœa and chancre, even if such a classification was sufficiently comprehensive, is highly objectionable; because the varieties of the same disease should not be designated by names having no analogy, as such a nomenclature cannot fail to perpetuate in the mind the erroneous supposition, that the maladies they express are essentially dissimilar, and not the result of the same cause. We must, therefore, endeavour to class venereal diseases in a manner more comprehensive, and more conformable to their natural relations." 57.

Here we see that Mr. Wallace openly and unhesitatingly maintains that syphilis and gonorrhœa arise from the same morbid poison. We will not repeat the arguments against this opinion, which have frequently been urged and never satisfactorily answered. We will merely observe that here, as on other occasions, Mr. Wallace begs the question. We have had no proof, unless the paragraphs on the transition states be considered such, of the common origin of sore and gonorrhœa, and we find no reply to the ordinary objections against such a theory. Mr. Wallace does indeed adopt an inverted argument, and urges in support of his opinion that gonorrhœa and chancre are sometimes combined, which is true—that they reciprocally produce each other, which is very doubtful—and that their influence on the constitution may be similar, which is directly opposed to common observation. The only acknowledged fact is, that sore and gonorrhœa are sometimes combined. When we reflect that females affected with sore and continuing to have connexion, as they constantly do, are very likely to contract gonorrhœa in addition, and, on the other hand, that these same females thus doubly infected may, in their turn, doubly infect, we repeat that when we consider this we see no reason to attach great weight to this the only general and indubitable fact adduced. The important and practical consideration opposed to the identy of the cause of syphilis and gonorrhœa, is the circumstance, that one, as a general rule, does evince a remarkable disposition to affect the system, and the other does not. But to proceed.

" When venereal diseases are contemplated with a view to their classification, a question naturally arises, whether any of the numerous and varied morbid actions or states produced by the direct application of the venereal poison, or any peculiar combination of these states, should be considered as being more particularly the legitimate effects of this poison?

The inoculation of the vaccine poison, like that of the venereal poison, produces numerous varieties of disease; yet we are in the habit of affirming, that one of these varieties deserves to be considered, in preference to the others, as the regular or legitimate vaccine disease. Should not the same law hold in regard to syphilis, which is, like vaccina, produced by a morbid poison? The answer is evident. The only question therefore is, which of the varieties of syphilitic disease should be considered the legitimate form, or primitive type? and which the irregular forms or degenerations? Or, in other words, what are the characters of syphilis, when regular in its progress, and what are the characters of those diseases which are to be considered its varieties? These questions will hereafter be answered in detail; but it may be now mentioned, that the disease, which in the following work is considered the original type or specific form, and of which all the other forms are viewed as degenerations, presents, when compared with these supposed degenerations, the following characters; which seem quite sufficient to stamp it as the specific or legitimate form of primary syphilis.

1. It exhibits in a combined and perfect state, and in a medium degree, that series of destructive and restorative actions, which are exhibited by the other forms of the same disease in an irregular manner, and without any defined proportions.

2. From it, as from a centre, the varieties branch out on either side, gradually decreasing in severity of destructive action, until they arrive at that state of disease, which consists in a morbid secretion without ulceration; and on the other hand as gradually increasing in severity, until they lapse into those forms of disease, which exhibit the most malignant ulceration or gangrene.

3. It is more easily propagated by artificial inoculation than any of the other forms of venereal disease. This is owing to its independence of contingent or accidental circumstances; and hence it is probably the form of primary syphilis which would invariably occur, if the action of the poison was uninfluenced by any such circumstances; whereas, we often fail in producing the other forms of venereal disease, because they are partly the result of accidental causes, which may not be present at the time of inoculation.

4. It is the most common form of syphilitic disease. The great frequency of discharges from the urethra does not contradict this observation, for these discharges may be easily accounted for by the facility with which any irritating secretion in the female, although not syphilitic, produces morbid secretions of the male urethra, which in our present state of knowledge we cannot perhaps always distinguish from such discharges as are caused by the syphilitic poison.

5. We shall hereafter find, that there is a form of venereal eruption or constitutional disease, which, according to its general relations or characters, as compared with other constitutional venereal eruptions, should be considered the legitimate form of eruption, upon the same principles that a certain form of primary disease is here considered to be the legitimate form; and this constitutional eruption exactly corresponds in its characters to the disease, which is assumed to be the regular form of primary disease. This analogy, between the primary and secondary disease, is conformable to that general law of similarity between primary and secondary symptoms, which governs the action of several morbid poisons; and may be received as an additional argument in proof that the form of disease under consideration is the specific form, or regular type of both primary and secondary syphilis when seated on the skin. It is not, however, to be supposed that the regular form of primary syphilis is uniformly or necessarily followed by the regular form of eruption. 59.

We know not how it is, but it so happens, that we cannot turn over a page of Mr. Wallace's book without finding debateable matter. We are usually averse to the arguing of general questions, but it is evident that, if we allow the opinions advanced and the premises laid down by Mr. Wallace to pass unquestioned now, we permit him to erect on a basis that we deem most fallacious his whole superstructure and system. We must say that there is a general laxity in Mr. Wallace's mode of reasoning, that renders it difficult to combat him. He seems to slip into a conclusion before the reader is aware that he is seriously aiming at it. What can be more vague than his analogy between the vaccine disease and the venereal? A vaccine vesicle which follows a certain progress is said to be regular and legitimate, because it is observed to follow such a course in the immense majority of instances, when obviously disturbing circumstances do not interfere. But Mr. Wallace's specific or legitimate sore is assumed to be such, because it is intermediate in degree between other affections, because it is more easily propagated by artificial inoculation, and because it is the most common form. The first reason will not bear examination—the second is more likely to be erroneous than correct, for gonorrhœa, which Mr. Wallace makes a form of syphilis, is probably more easily communicated than any kind of sore, and the experiments on the inoculation of venereal ulcers are obviously imperfect—and the third reason is also inconclusive, as, setting aside the greater frequency of gonorrhœa, it is an assumption to assert that, because one kind of sore is rather more common than another, it must necessarily be the true and specific type.

Mr. Wallace now passes to the description of the particular forms of sore, to the description, in fact, of the venereal disease as it presents itself in nature. However we may differ from Mr. Wallace in his general views, we shall have occasion to bear ample testimony to his accuracy of observation and fidelity of detail. We cannot, in this number, proceed with the present review, but in our next we shall have the pleasure of presenting an analysis of the remainder of the volume. Mr. Wallace will find that none can be more willing than ourselves to do justice to his labours.

